# Bacteria increase arid-land soil surface temperature through the production of sunscreens

**DOI:** 10.1038/ncomms10373

**Published:** 2016-01-20

**Authors:** Estelle Couradeau, Ulas Karaoz, Hsiao Chien Lim, Ulisses Nunes da Rocha, Trent Northen, Eoin Brodie, Ferran Garcia-Pichel

**Affiliations:** 1 School of Life Sciences, Arizona State University, Tempe, Arizona 85287, USA; 2 Earth and Environmental Sciences, Lawrence Berkeley National Laboratory, Berkeley, California 94720, USA; 3 Environmental Genomics and Systems Biology, Lawrence Berkeley National Laboratory, Berkeley, California 94720, USA; 4 Department of Environmental Science, Policy and Management, University of California, Berkeley, California 94720, USA

## Abstract

Soil surface temperature, an important driver of terrestrial biogeochemical processes, depends strongly on soil albedo, which can be significantly modified by factors such as plant cover. In sparsely vegetated lands, the soil surface can be colonized by photosynthetic microbes that build biocrust communities. Here we use concurrent physical, biochemical and microbiological analyses to show that mature biocrusts can increase surface soil temperature by as much as 10 °C through the accumulation of large quantities of a secondary metabolite, the microbial sunscreen scytonemin, produced by a group of late-successional cyanobacteria. Scytonemin accumulation decreases soil albedo significantly. Such localized warming has apparent and immediate consequences for the soil microbiome, inducing the replacement of thermosensitive bacterial species with more thermotolerant forms. These results reveal that not only vegetation but also microorganisms are a factor in modifying terrestrial albedo, potentially impacting biosphere feedbacks on past and future climate, and call for a direct assessment of such effects at larger scales.

## Abstract (web summary)

Soil surface temperature, which affects many biogeochemical processes, depends on soil albedo. Here, Couradeau *et al.* show that some cyanobacteria can increase the temperature of arid-land soil surface by as much as 10 °C through the accumulation of a sunscreen metabolite.

## Graphical abstract

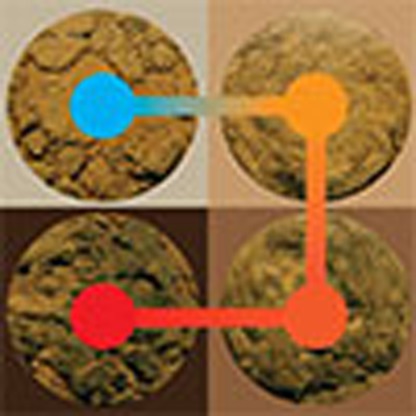

Surface soil temperatures are a function of the downwelling electromagnetic radiation and of the albedo (reflectivity) of the soil surface^1^. Surface temperature is known to influence a variety of ecological and biogeochemical soil processes, from plant germination and microbial activity, to evapotranspiration and gas emissions^1^,^2^. In temperate climates, biological factors such as plant cover, do in turn modify the albedo, thus influencing local temperatures^3^,^4^, but plant effects are of little relevance in arid lands, where vegetation cover is restricted or negligible.

Much of the arid soil surfaces can be populated by cryptic photosynthetic assemblages known as biological soil crusts (or biocrusts)^5^,^6^, which are known to impart stability against erosion^7^, to modify the hydrological properties of soils^8^, and to contribute significantly to arid land fertility^9^. These communities also represent the major type of ecosystem on the continents before the advent of land plants in the Devonian^10^. When sufficiently developed, biocrusts can reduce the albedo of the soils they cover^11^, and in some instances their development has been linked to concurrent increases in soil surface temperature^12^,^13^,^14^. The mechanism by which biocrusts decrease albedo, the extent of the phenomenon and the consequences on soil temperature and biological processes, if any, remain to be studied.

To explore these issues, we assessed soil surface temperature, pigment content and microbial composition in biocrusts of varying successional maturity, as they transitioned during development from early and largely cryptic, to mature and conspicuously dark, in a single site from the Colorado Plateau of North America. We found that some cyanobacteria warm the soil surface by as much as 10 °C through the production of scytonemin, an alkaloid sunscreen pigment that strongly absorbs solar radiation and dissipates this energy as heat. This warming is not without consequences for the soil microbial communities, as the sunscreen-driven warming correlated with a replacement of the keystone heat-sensitive species *Microcoleus vaginatus* by the more heat-tolerant *M. steenstrupii*.

## Results and Discussion

**Figure 1:**
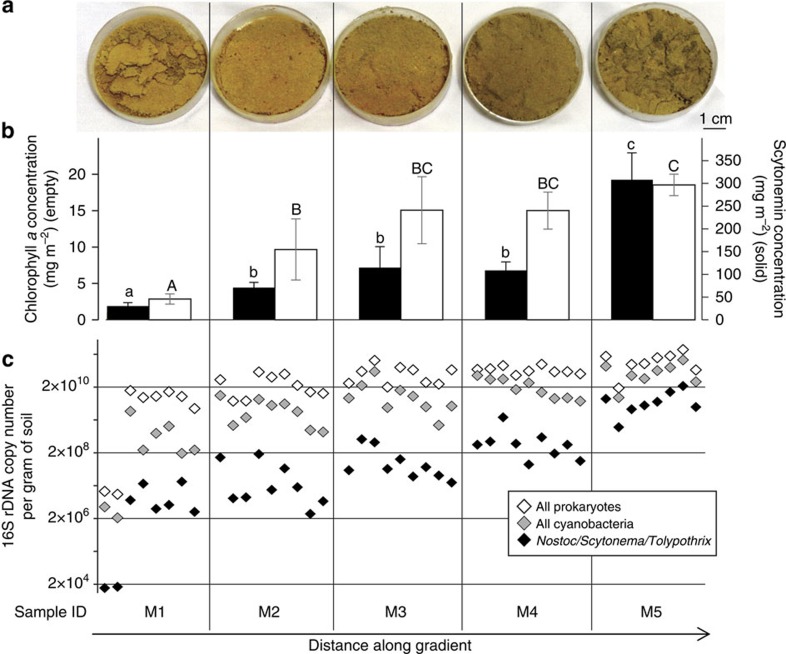
Characterization of cyanobacteria dominated biological soil crusts gradients of maturity (space for time). (**a**) Aspect of typical samples in each category of the gradient (one replicate) showing increasing darkness with distance/maturity. (**b**) Scytonemin (solid) and chlorophyll *a* (empty) areal concentrations. The error bars are ±s.d. (*n*=3). Different letters indicate statistically different means (LSD *post hoc* test *P*<0.05). (**c**) 16S rDNA copy number per gram of soil assessed by qPCR, where each gradient level comprises 8–9 replicates, for all prokaryotes (empty), all cyanobacteria (grey) and heterocystous cyanobacteria (genera *Nostoc*, *Scytonema* and *Tolypothrix*) (solid).

Ecological succession in biocrusts in these region starts with colonization by pioneer, soil-stabilizing, motile filamentous cyanobacteria^15^, and is followed by the eventual establishment of surface-bound, sessile heterocystous cyanobacteria^16^. During the process, overall microbial biomass increases steadily, and eventually the surface darkens conspicuously^16^,^17^. We used this progressive darkening to guide the collection of samples of different maturity from a single site, North of Moab, Utah. In a first sample set (Fig. 1) we followed an apparent gradient of maturity that had developed with distance from a small wash (space for time) using repeated samples as replicates. The samples were characterized *post hoc* with respect to major pigments (including chlorophyll *a* as a proxy of photosynthetic biomass), which increased across the gradient from very low concentrations corresponding to virtually uncrusted soils, to those that could be deemed as fully developed in comparison with published biocrust surveys^18^. We also determined microbial biomass, and the biomass attributable to relevant microbial groups by absolute counts of relevant 16S rRNA gene copies (Supplementary Table 1 and Fig. 1c) in this set. Other sets of samples were selected to study temperature and albedo as a function of pigment content (Supplementary Table 2 and Fig. 1a,b).

**Figure 2:**
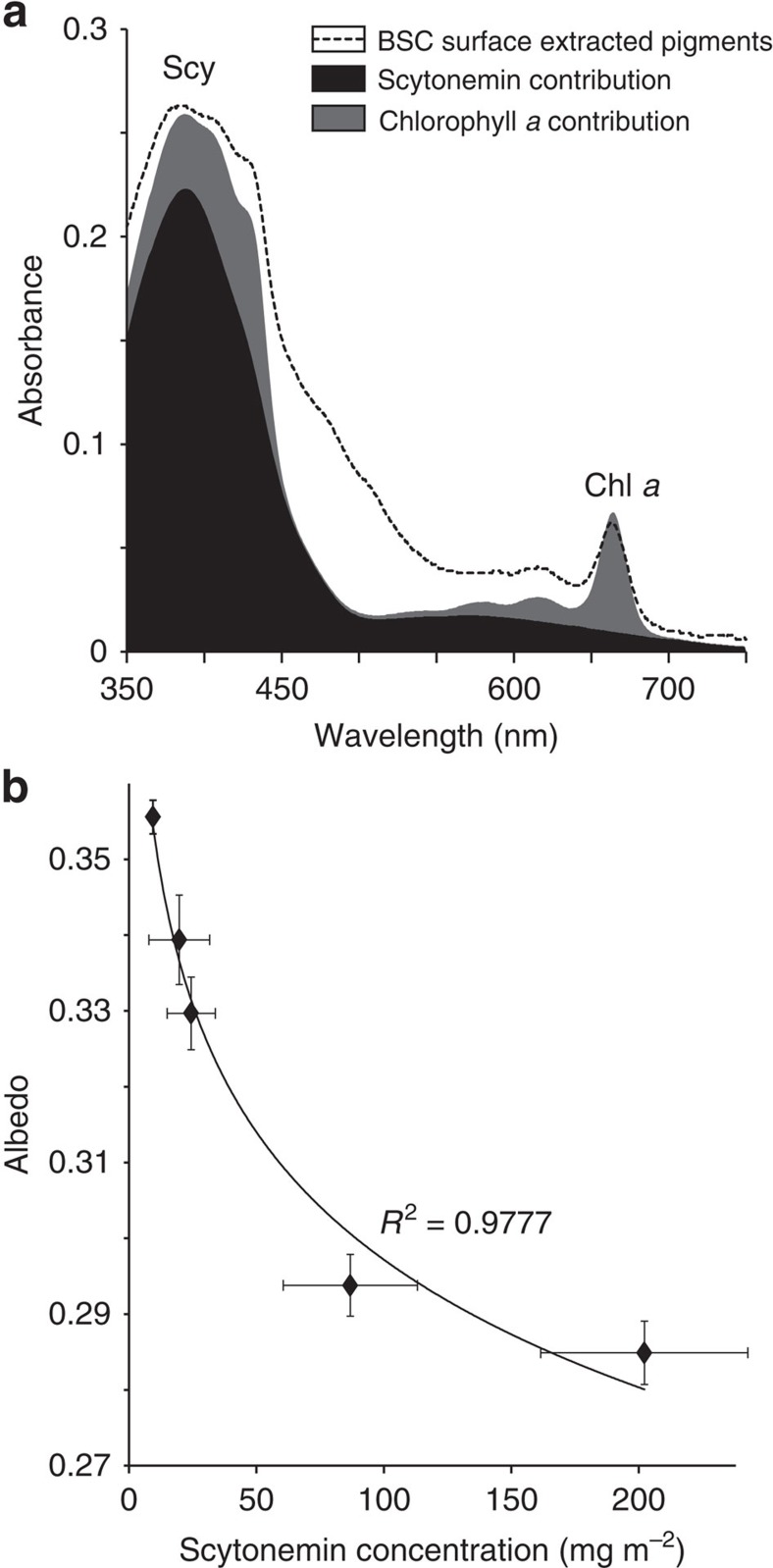
Scytonemin as major contributor to light absorption and its relationship with biocrust albedo. (**a**) Absorbance spectrum of lipid-soluble pigments in the surface biomass of a dark crusts (as acetone extract; dashed line), with calculated contributions to the total absortion by scytonemin (Scy, black) and chlorophyll a (Chl *a*, grey). (**b**) Logarithmic (Ln) relationship between measured albedo and areal scytonemin concentration in a series of crust samples of varying maturity. BSC stands for Biological Soil Crust.

Given that all samples shared a common sedimentary parent material (mostly quartz sand) and were flat, the conspicuous differences in albedo were likely due to the effect of microbial pigment accumulation rather than to particle size or surface roughness^19^. The contribution of the latter may be of importance in later successional stages such as the more developed pinnacled dark biocrusts. Absorbance spectra of surface-bound bacterial biomass in the visible range (Fig. 2a) indicated that the large majority of the pigments contributing to differential albedo were chlorophyll *a*, small amounts of carotenoids and the cyanobacterial sunscreen scytonemin^20^. No infrared (IR) absorbing pigments (like bacteriochlorophylls) were measurable. An integration of relative absorption over the visible wavelength range, showed that the sunscreen alkaloid scytonemin contributed most to absorption in mature samples, 6±0.8 (*n*=6) times more than chlorophyll *a* to visible photon absorption on the crust surface. It seems clear that any albedo reductions associated with biocrust development are due largely to the accumulation of scytonemin. Surface albedo is also influenced by the presence of microbially secreted exopolymeric substances, their removal results into a decreased albedo^21^. However, if the accumulation of exopolymeric substances with maturity increases the albedo, it plays against the trend described here, which in fact reinforces the hypothesis of scytonemin accumulation being the major force driving the observed albedo decrease with maturity. Indeed, measurements of the crust albedo under standard illumination conditions showed that it was predictably an inverse logarithmic function of scytonemin pigment content (Fig. 2c). Among the various cyanobacteria that colonize biological soil crusts^22^, only the late-stage group of nitrogen-fixing, heterocystous cyanobacteria (in the order *Nostocales*) produce the compound. Indeed, the abundance of scytonemin-producing *Nostocales,* based on the absolute copy number of 16S rRNA genes (Fig. 1c), tracked the increase in scytonemin itself.

**Figure 3:**
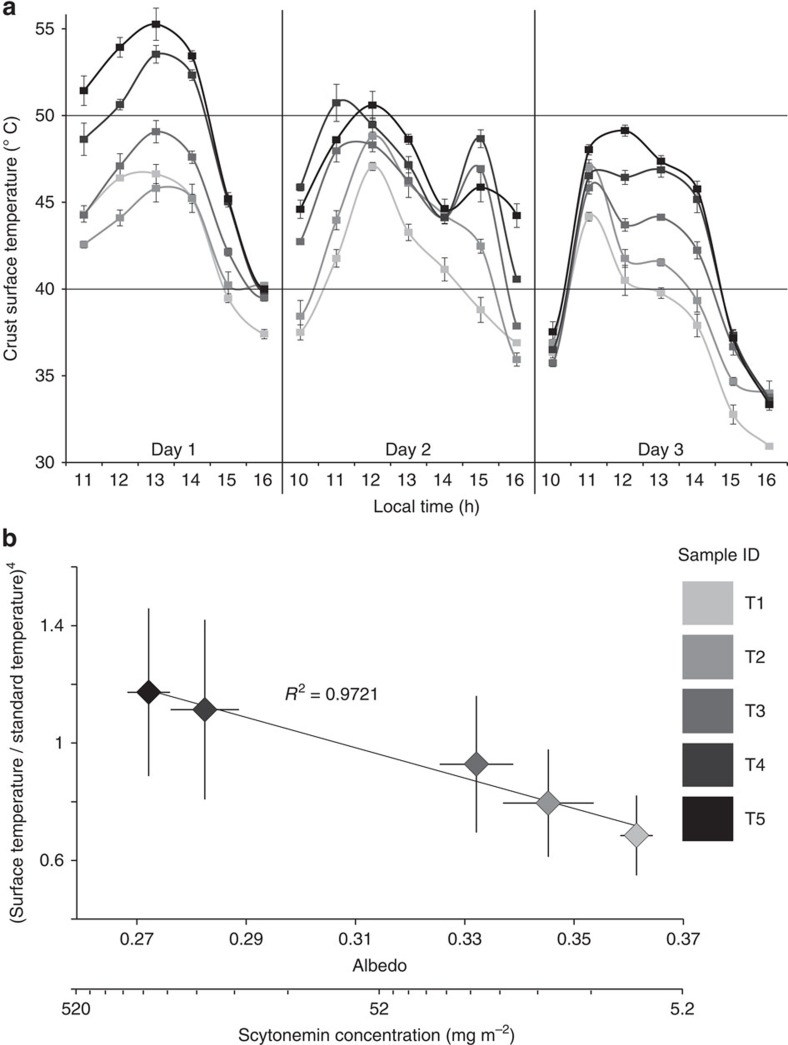
Soil surface temperature evolution around midday in discrete soil crust samples and its relation to albedo and scytonemin content variations. (**a**) Surface temperature evolution in samples exposed to outdoor irradiance around noon during three consecutive days in autumn. (**b**) Relationship between albedo and differential warming. The fourth power of the ratios between a biocrust surface temperature and the temperature attained under equal conditions by a standard probe, are predictably (see Methods) a linear function of albedo. Scytonemin concentration was directly determined in each sample and used to arrive at albedo using the relationship in Fig. 2.

In order to test the effects of scytonemin accumulation on biocrust temperature, we performed direct measurements on a subset of five selected samples. These were exposed to natural autumnal sunlight around the brightest part of the day on a rooftop in Tempe (Arizona) (Supplementary Fig. 1c). Global visible radiation during those days peaked at 935 W m^−2^, which corresponds to intensities reached at midday all year long in their original locality (National Solar Radiation Database). We observed clear temperature differences among samples, with the hottest temperatures at any one time attained in the darker samples, and with maximal temperature differences between the two extremes of crust maturity reaching 10 °C around solar noon (Fig. 3a). In addition to receiving the same solar radiation flux, no significant differences in heat transfer between samples and the environment are likely among our samples, so we conclude that the observed temperature differences result from changes in albedo. As can be theoretically predicted if albedo drives the differences, the fourth power of the ratio between the temperatures attained by a given sample against that attained by a standard probe of fixed albedo should be a decreasing linear function of the sample's albedo, and, thus, a saturating function of the concentration of the major absorber (see Methods). We included a temperature probe adjacent to the samples in Fig. 3a as a standard, and show this relationship in Fig. 3b. At low concentrations of scytonemin (that is, >50 mg m^−2^) the relationship between pigment concentration and differential warming (Supplementary Fig. 2) can be simplified to a linear relationship (which translates to an average temperature differential of 0.16 °C per mg of scytonemin per m^2^). However at scytonemin concentrations typical of the upper range in our survey (that is, >100 mg m^−2^), warming reached a saturating effect, and further increases in pigment elicited a much reduced warming effect. Maximal biocrust concentrations of scytonemin reported from the literature and from our own surveys very rarely exceed 700 mg m^−2^ (ref. 18). Because of this, and because the ratio of equilibrium temperatures between sample and probe does not depend on downwelling light intensity, but only on albedo (see Methods), we do not expect that the soil warming effect by scytonemin relative to bare ground will exceed by much the values reported here. The absolute temperatures reached by a biocrusted soil, however, will depend on overall heat transfer coefficients and ambient temperatures, so that biocrusts in low latitudes with already hot climates may potentially reach biologically deleterious temperatures, whereas for biocrusts in cold climates, that is, in polar settings or during winter months when activity is limited by temperature, the warming can be expected to be largely beneficial to biological entities.

**Figure 4:**
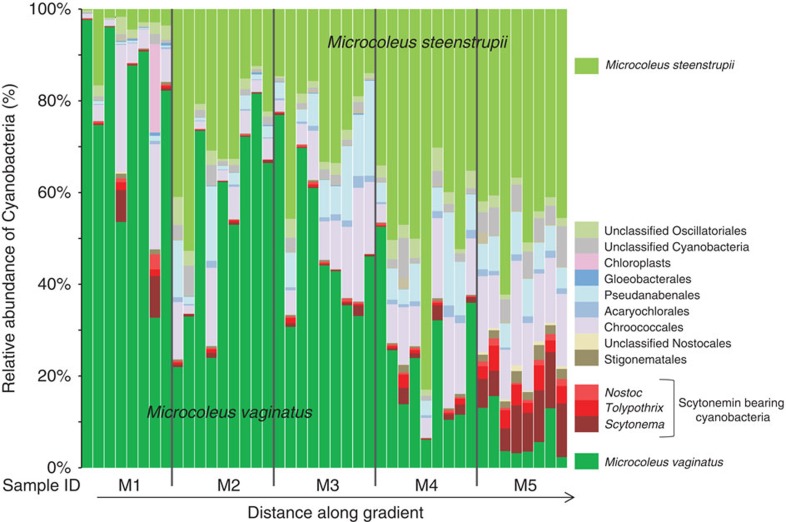
Cyanobacterial shifts in community composition along a maturity gradient. Relative distribution of cyanobacterial phylotypes, defined on the basis of SSU rRNA gene sequences, along crust maturity gradients (data from the same subset of samples as in Fig. 1). Each gradient level (M1–M5) comprises 8–9 replicate samples, corresponding to those in Fig 1. A clear replacement of dominance in thermosensitive *Microcoleus vaginatus* by thermotolerant *M. steenstrupii*^22^ as dominant microbe coincides with the relative increase of sunscreen bearing cyanobacteria.

A question that follows naturally from the previous findings is if the differential warming by sunscreen pigments we measured does in fact affect biological or biogeochemical processes in the crusts and soils. In a recent contribution relating biological soil crusts community composition to temperature, it was shown that continental-scale temperature differentials (yearly averages) of smaller magnitude than those measured here (∼5 °C versus 10 °C, respectively) were sufficient to induce a clear biogeographic shift between two keystone biocrust microbes^22^, one type (*M. vaginatus*) being much more thermosensitive than the other (*M. steenstrupii*), whereas other environmental parameters failed to explain their biogeography. These two organisms thus represent a form of biological sensor for an integrated response to warming. We determined changes in community structure along the gradient of maturity described in Fig. 1 with deep 16S rRNA gene surveys using next-generation sequencing (Fig. 4). Focusing these analyses on the phototrophs (cyanobacteria and algae), we could in fact observe a clear replacement in the dominant cyanobacterial phylotypes, assignable to the motile, bundle forming, filamentous cyanobacteria^15^,^23^: *M. vaginatus* dominated in light, incipient crusts subject to the least differential warming, while *M. steenstrupii* became more abundant in the darker, more mature ones that reached maximal differential warming. As discussed previously^15^,^22^, *M. vaginatus* constitutes a very well defined monophyletic group and has a coherent, cold-loving phenotype, whereas, *M. steenstrupii,* which encompasses a much more diverse group of cyanobacteria (Supplementary Fig. 3), is more thermotolerant. Thus soil warming due to microbial sunscreens, has an equivalent biological manifestation at the local (meter) scale as seen for climate variability at the continental scale^22^.

It is clear from these results that the role of microbes in soil warming and its potential for impact on species distributions and biogeochemical cycles should be considered in studies of arid and semi-arid systems at local and landscape scales. Arid lands where biocrusts feature prominently, cover 41% of the Earth's land surface^5^; such crusts are thought to have dominated terrestrial ecosystems from much of the Proterozoic^10^, where the production of UVA suncreens such as scytonemin would have been of major fitness value after the oxygenation of the atmosphere^24^. For these reasons, microbial effects on land surface albedo may have global scale repercussions historically and in the present, and should be evaluated in models of planetary radiation budgets as a new twist of biosphere-climate feedback interactions. This may shed some light into the apparent inconsistency in temperature changes recorded in arid lands. Contrary to model predictions^25^, temperature has been shown to increase when removing vegetation there^26^,^27^, an apparent paradox that could potentially be explained by biocrust colonization. Based on estimates of the global biomass of cyanobacteria in soil biocrusts^28^, one can easily calculate that there must currently exist about 15 million metric tons of scytonemin at work, warming soil surfaces worldwide.

## Methods

### Site and sampling description

Biocrust samples were collected using petri dishes (6 cm diameter, 1 cm depth) in the Green Butte Site (38°42′53.9′′N, 109°41′34.6′′W, Moab, Utah, USA). Samples were transported air-dry, and maintained in the dark under an atmosphere in equilibrium with LiCl desiccant until experimentation. The samples belong to three different sets (M, T and A set) used for different assays. Subset M was sampled in July 2011 as a space-for-time maturity gradient in the field, with four replicate dishes taken at the same distance along the main axis. Three sets of biological replicates of the M series were used for the assessment of the microbial community using molecular methods. Each gradient level was sampled dry, then wetted and resampled twice over a period of 24 h. This was because microbes in biocrusts suffer a DNA damage while dry, but are able to repair their DNA in a few hours after wetting^29^, so we were expecting a better reading of the microbial community DNA upon wetting, but did not want to allow them to grow differentially by resampling later than 24 h. After bioinformatics data processing and taxonomic assignment of the 16S rDNA recovered, we tested the effect of the wet-up treatment on the cyanobacterial community structure; no significant effect was found through repeated measures analysis of variance (Supplementary Table 2), so we considered the nine DNA extracts within one gradient level as equivalent replicates. The last set of the M series was used to measure pigment content in the set. The other two sets of samples (A and T) were collected in October 2012 and picked so that they would span roughly the same maturity gradient as the M series, according to their pigment concentration. A and T series were used to measure albedo and temperature effects, respectively, as a function of pigmentation.

### Nucleic acids purification

We extracted total genomic DNA for each of the three replicates, each of the five gradient levels and each of the three time point (that is, 45 samples total). The detailed protocol of the nucleic acids extraction is published in ref. 29. Briefly, we used CTAB (5% CTAB, 0.25 M phosphate buffer pH 8.0, 0.3 M NaCl), aluminum ammonium sulfate (0.1 M), 30% (w/v) polyethylene glycol 6,000 and 1.6 M NaCl precipitations steps followed by a separation of DNA and RNA using the AllPrep DNA/RNA Mini Kit (Qiagen Inc, Valencia, CA, USA) and the QIAcube (Qiagen Inc, Valencia, CA, USA) following manufacturer's instructions.

### SSU rDNA copy number determination by qPCR

After Qubit (Life Technologies, New York, USA) determination of total DNA concentration, the copy number of SSU rDNA per ng of DNA has been assessed using a qPCR (real-time PCR) with the general SSU rDNA primer set: 338F 5′-ACTCCTACGGGAGGCAGCAG-3′, 518R 5′-GTATTACCGCGGCTGCTGG-3′. The PCR has been done using the Sso Fast mix (Bio-Rad, Hercules, CA, USA) under these conditions: an initial denaturation phase (2 min at 98 °C), followed by 40 cycles (denaturation at 95 °C for 10 s, annealing at 55 °C for 30 s, followed by the melting curve acquisition (increasing temperature from 55 to 95 °C at 0.5 °C s^−1^ speed). We performed each PCR reaction in triplicate and averaged the total number of SSU rDNA per sample.

### SSU rDNA libraries construction and Illumina sequencing

We described the microbial diversity using prokaryotic SSU rDNA library sequencing. This technique has been shown to be more accurate than shotgun metagenomic to accurately describe the dominant taxa in biocrust^30^. We amplified a total of 10 ng of genomic DNA per sample. We used general prokaryotic primers targeting the V4 hypervariable region of the SSU rDNA: forward 515F 5′- GTGCCAGCMGCCGCGGTAA-3′, reverse 806R 5′- GGACTACHVGGGTWTCTAAT -3′. Each sample genomic DNA was amplified with a reverse primer customized with an adapter, a primer pad, a specific Golay barcode^31^ and a linker. The PCR was done in triplicate for each sample and PCR products have been pooled afterwards, using the Takara ExTaq (Millipore, Billerica, USA) and reagents under these conditions: an initial phase of denaturation (4 min at 94 °C), followed by 24 cycles (denaturation at 94 °C for 20 s, annealing at 50 °C for 30 s, extension at 72 °C for 42 s), followed by a final extension phase (10 min at 72 °C). We cleaned the PCR products using 0.1% Seramag beads in buffer solution: after incubation on a magnetic plate for 2 min, the PCR supernatant was discarded, the beads were washed twice with 80% ethanol and the PCR products were eluted within 50 μl of 1 × TE. We analyzed the cleanliness of twelve samples randomly picked with the Bioanalyzer (Agilent Technologies, Wilmington, USA). We quantified the concentration of PCR products by Qubit and pooled them to achieve a final amount of 25 ng of DNA per sample in the library. The pooled DNA library concentration was assessed using the KAPA SYBR FAST qPCR Kit following manufacturer's instruction (KapaBiosystems, Boston, USA). After dilution and denaturation, we loaded a mix of 60% library, 40% PhiX (as recommended by Caporaso *et al.*^32^) on to the Miseq Illumina (500 cycles) cartridge for a final concentration of 20 pM DNA. The sequencing was performed on MiSeq using paired ends sequencing and default chemistry.

### Bioinfomatic analyses and phylogeny

We demultiplexed the FastaQ file generated by the MiSeq Illumina workflow using CASAVA 1.8.1 (Illumina, San Diego, USA). We trimmed the sequences for a Phred quality score superior to 30 (Q>30) using Trimmomatic (ref. 33). Then, the sequences were paired end assembled using PANDAseq (ref. 34) and converted to fasta using prinseq-lite.pl script (ref. 35). The data set was processed with Qiime (ref. 36) through the ‘pick_otus_through_otu_table.py' pipeline to pick OTUs (Operational Taxonomic Units) with a cut-off of 97% sequence similarity. The parameter file used is included as Supplementary Software. The OTUs were filtered, so that only the ones with at least one sequence read shared by at least two samples were kept. The filtered OTU table was used for taxonomy analyses and calculation of diversity indices (Supplementary Table 2).

The maximum-likelihood phylogenetic reconstruction was performed using TREEFINDER (ref. 37) under a general time reversible (GTR) and a four-category discrete approximation of a Γ distribution. Bootstrap values were inferred from 1,000 replicates. The sequence dataset used for the reconstruction was first aligned with MAFFT (ref. 38) and then manually checked and trimmed using the MUST package^39^.

### Chlorophyll a and scytonemin measurement

Pigments were extracted from three pieces of known surface (each sample surface was measured individually, on average the samples used were 189±58 mm^2^) of any given biocrust sample in a 1:10 volume ratio of soil: 90% acetone at 4 °C for 24 h. Absorbance spectra were recorded on a UV-Visible Spectrophotometer (Shimadzu UV-1601) between 350 and 750 nm. Pigments concentration were calculated using trichromatic equations developed by Garcia-Pichel and Castenholz^20^ to deconvolute each pigment's contributions to absorbance. The extinction coefficients (ɛ) used were 89.7 l g^−1^ cm^−1^ for chlorophyll *a* (ref. 40) and 112.6 l g^−1^ cm^−1^ for scytonemin^41^. An analysis of variance revealed statistically significant differences of pigment concentration between samples, posthoc analyses results on paired samples (LSD) are reported on the Fig. 1b. In order to calculate relative absorption of visible light by scytonemin and chlorophyll *a*, pure scytonemin was separated from other pigments in an extract using thin layer chromatography (TLC) in a 9:1 mixture of chloroform: methanol^20^. Pure scytonemin and pure chlorophyll *a* (Sigma-Aldrich, St Louis, USA) absorbance spectra in 90% acetone were recorded on a UV–visible spectrophotometer (Shimadzu UV-1601) between 350 and 750 nm. The values of the extinctions coefficients were extrapolated for the whole spectra and we calculated the integration of the ratio of *ɛ*_scy_/ *ɛ*_chla_ between 350 and 750 nm, for a final result of 1.5.

### Calculation of the absorbance ratio between major pigments from biocrust surface

Using the following Beer Lambert law, we were able to calculate the absorbance ratio of scytonemin (A_scy_) and chlorophyll *a* (A_chla_) in the visible spectrum (350–750 nm), depending only on the pigments concentration (C_scy_ and C_chla_) for any given sample. The absorbance (A) is unitless, the extinction coefficients (*ɛ*) are in l g^−1^ cm^−1^ and the concentrations (C) are in g l^−1^. The path length (which otherwise would be in cm) was omitted in the development of the absorbance because this term is equal in the numerator and in the denominator so it cancels itself out. The overall ratio of absorbance is unitless.


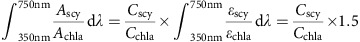


### Albedo measurement

Supplementary Fig. 1a shows the set-up used to assess the albedo of samples. Because we were interested in the radiative equilibrium temperature, we approximated the plane albedo^42^. To estimate the plane albedo, the light source has to be fixed and constant and the reflected light has to be measured ideally at all angles (accounting for a total hemisphere), which we simplified here to a set of 5 angles. The samples were illuminated by a halogen goose-neck lamp placed at a 45° angle from the sample surface. A blue multi-coated HOYA HMC 80A filter was added in the light path to balance the light energy and better match the solar spectrum. The radiance reflectance was measured with a Li Cor LI-250 light meter in five different elevation angles (see Supplementary Fig. 1a, angles *c1-c5*) regularly spread between 0° and 90° from the sample surface and constrained in its acceptance angle to ±10°. Eight independent measurements for each angle were averaged in order to assess the reflectivity of a sample. The data acquired from the horizontal angle (*c5*) were not used because this angle was receiving direct light from the light source. Two reflectivity standards (0.02 and 0.5 reflectance) were measured the same way so we could control the accuracy of the set-up and convert the reflectivity values of the samples into plane albedo values.

### Surface temperature assay

A set of samples was used to assess differential warming. Samples were placed on an open area, during three clear days in Tempe, AZ. The surface temperature of each sample was measure every hour between 1,100 and 1,600 hours by a fixed 566 Fluxe IR Thermometer gun (Fluke corporation, Everett, WA, USA) (Supplementary Fig. 1b). The thermometer integrated the temperature of a 2 cm diameter area centred on the dish that contained the sample. We recorded four independent measurements at each time-point. A grey temperature probe (Amprobe TH-1) was placed on the bench next to the sample, in the same conditions of illumination.

### Albedo–temperature relationship

The total energy received by a body (*E*_in_) can be expressed as the global horizontal irradiance (*I*_0_) times the absorption of the body given by (1−*a*) where *a* is the albedo (equation 1; ref. 1). The total energy emitted by a body (*E*_out_) is given by the Stefan-Boltzmann law (equation 2; ref. 1), where *ɛ* is the emissivity coefficient, *σ* is a constant and *T* is the surface temperature. At equilibrium, *E*_in_=*E*_out_ (equation 3). In the range of temperature reached here, most of the thermal radiation happens in the IR range. Since the pigments do not absorb in the IR, the emissivity of the samples will not depend on their color in the visible, and it is reasonable to assume that they will have similar emissivity values, being composed of the same background material. In our experiment, together with the surface temperature of the samples (Bsc), we also measured the temperature reached by a grey probe that was placed next to the samples (Std), so that both were receiving the same global horizontal irradiance (*I*_0_). According to equation 4 the ratio between the biocrust sample temperature (*T*_Bsc_) and the probe temperature (*T*_Std_) raised to the fourth power is a linear function of albedo, as in equations 4 and 5, where *A* is a constant that encompasses all constant terms in (4). We sought to match this relationship to the ratio of temperatures in our data set.


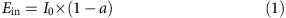



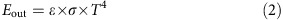



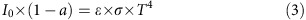



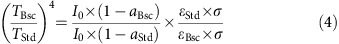



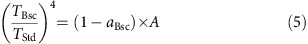


## Additional information

**Accession codes:** The SSU rDNA sequences have been deposited in the NCBI Bioproject database under accession number PRJNA299730.

**How to cite this article:** Couradeau, E. *et al.* Bacteria increase arid-land soil surface temperature through the production of sunscreens. *Nat. Commun.* 7:10373 doi: 10.1038/ncomms10373 (2016).

## Supplementary Material

Supplementary Figures and Supplementary Tables Supplementary Figures 1-3 and Supplementary Tables 1-2

Supplementary Software Parameter file used for the treatment of Illumina 16S rDNA libraries data in the qiime pipeline.
