# Supplementary material for: Bacteria increase arid-land soil surface temperature through the production of sunscreens
Source: Nat Commun. 2016 Jan 20;7:10373. doi: 10.1038/ncomms10373 (PMC4735820; doi:10.1038/ncomms10373)
Supplement: Supplementary Figures and Supplementary Tables — Supplementary Figures 1-3 and Supplementary Tables 1-2 [file ncomms10373-s1.pdf]

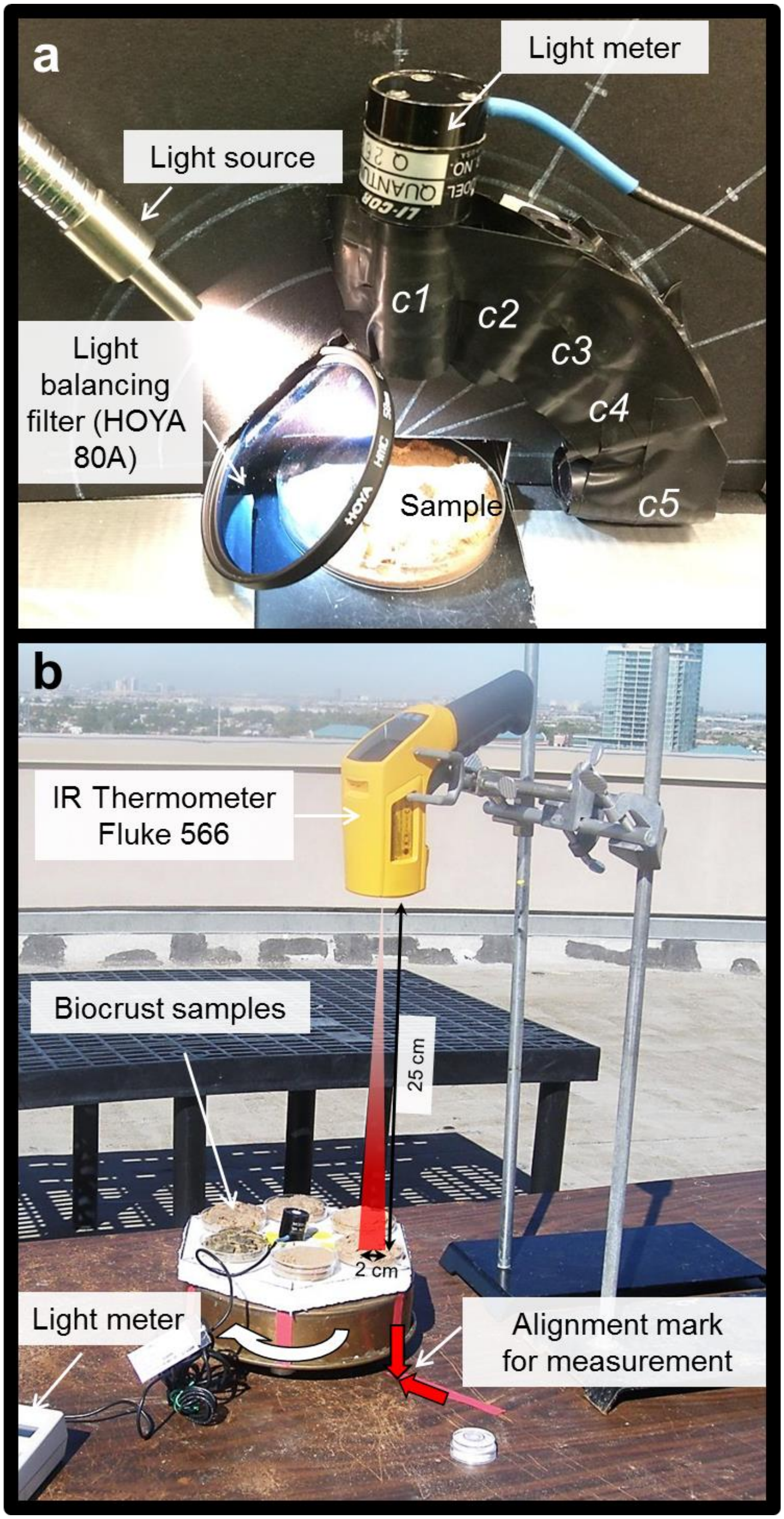

a
Light meter
Light source
Light balancing filter (HOYA 80A)
c1
c2
c3
c4
c5
Sample
b
IR Thermometer Fluke 566
Biocrust samples
25 cm
2 cm
Light meter
Alignment mark for measurement

Supplementary Figure 1: Experimental set-up used for the assessment of biocrust albedo and surface temperature.

(a) Experimental set-up used for the assessment of biocrust albedo. Samples are illuminated with a focused tungsten goose-neck source corrected with a day light filter at a 45 °C elevation angle. Radiance reflectance is measured at 5 angles (*c1-c5*) using a visible light meter using comparison with reflectance standards (100% and 50%). Albedo was defined as the reflectance averaged over all angles. (**b)** Experimental set-up used for the surface temperature survey. The samples are on a rotating sample holder, while the IR thermometer is fixed, 25 cm from the surface of the samples. Samples are aligned to a mark so that the IR gun points to the center of the sample, integrating the surface temperature of a 2 cm diameter area. The temperature probe (Amprobe TH-1) was placed on the table next to the samples at the time of measurement.

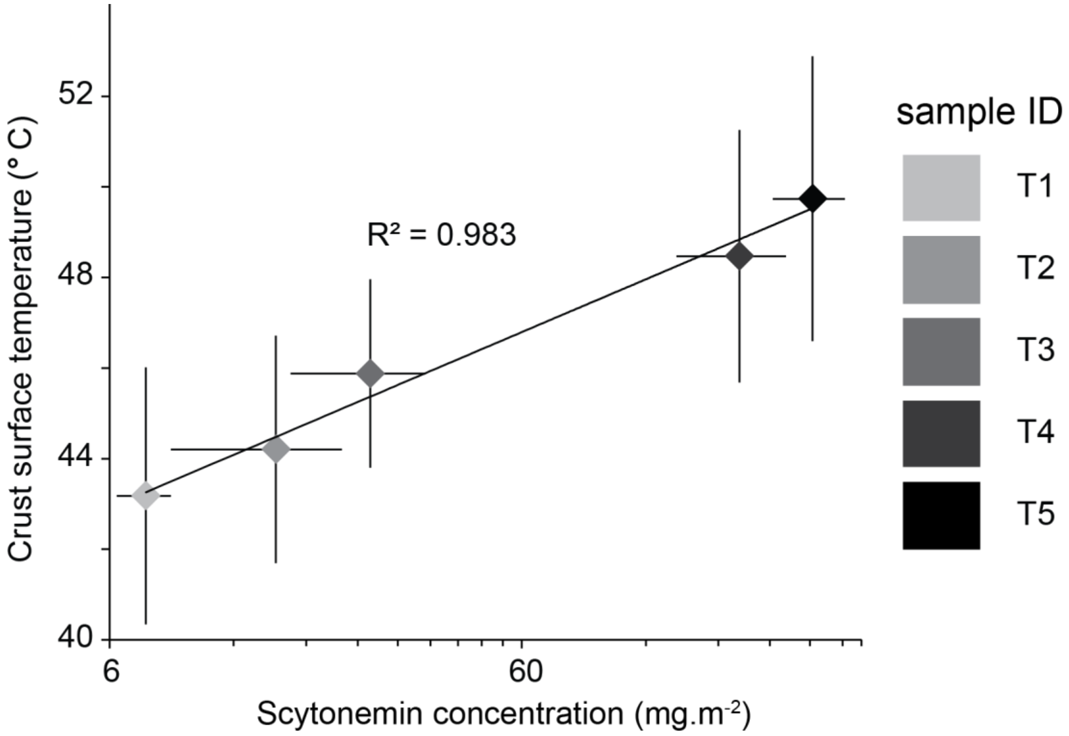

Supplementary Figure 2: Logarithmic (Ln) relationship between scytonemin concentration and Biocrust surface temperature.

At low concentrations of scytonemin (i. e. < 50 mg m^{-2}), the relationship can be simplified to a linear relationship (with an average temperature differential of 0.16 ºC mg [scytonemin] m^{-2}).

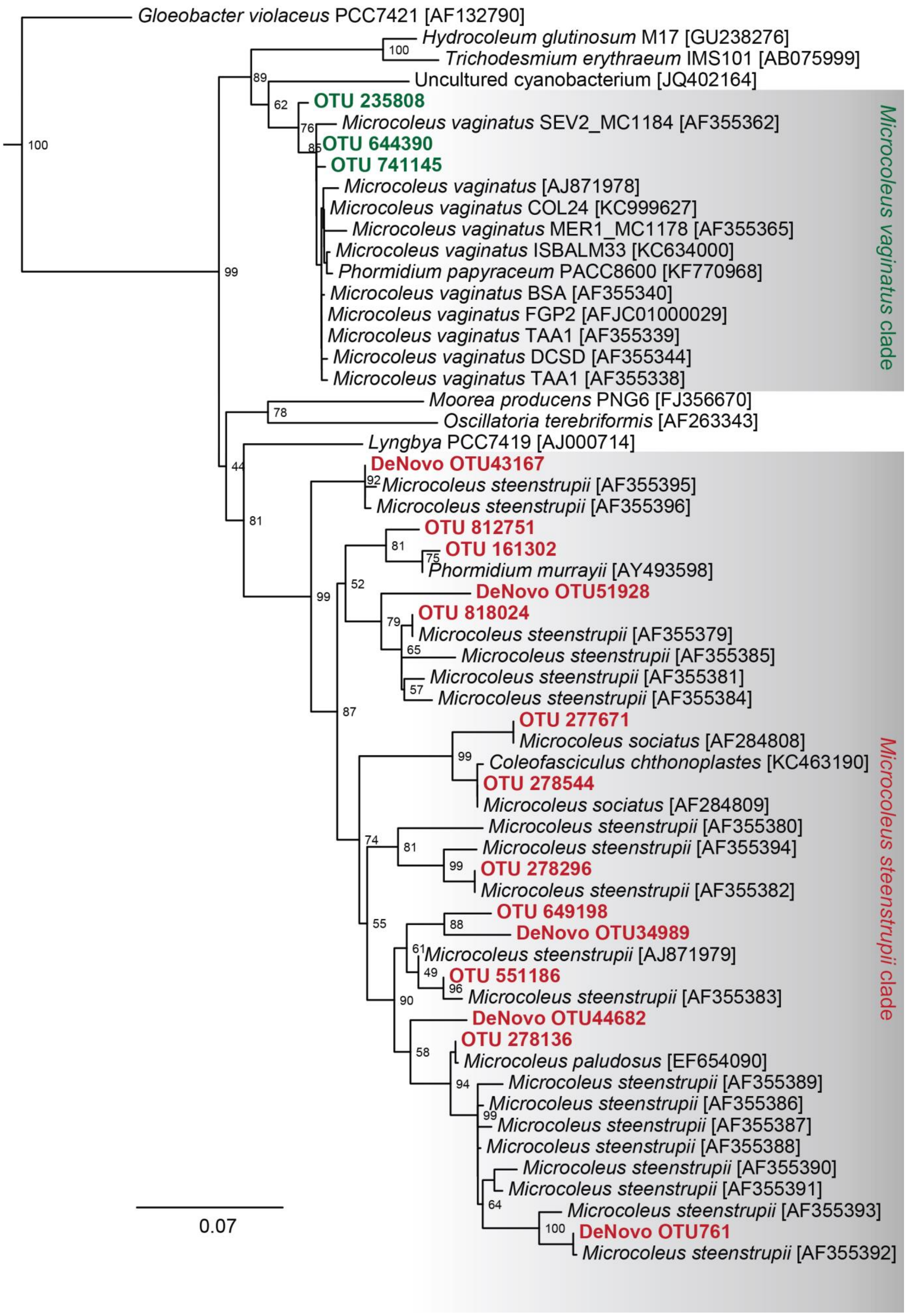
Gloeobacter violaceus PCC7421 [AF132790]
Hydrocoleum glutinosum M17 [GU238276]
Trichodesmium erythraeum IMS101 [AB075999]
Uncultured cyanobacterium [JQ402164]
OTU 235808
Microcoleus vaginatus SEV2_MC1184 [AF355362]
OTU 644390
OTU 741145
Microcoleus vaginatus [AJ871978]
Microcoleus vaginatus COL24 [KC999627]
Microcoleus vaginatus MER1_MC1178 [AF355365]
Microcoleus vaginatus ISBALM33 [KC634000]
Phormidium papyraceum PACC8600 [KF770968]
Microcoleus vaginatus BSA [AF355340]
Microcoleus vaginatus FGP2 [AFJC01000029]
Microcoleus vaginatus TAA1 [AF355339]
Microcoleus vaginatus DCSD [AF355344]
Microcoleus vaginatus TAA1 [AF355338]
Microcoleus vaginatus clade
Moorea producens PNG6 [FJ356670]
Oscillatoria terebriformis [AF263343]
Lyngbya PCC7419 [AJ000714]
DeNovo OTU43167
Microcoleus steenstrupii [AF355395]
Microcoleus steenstrupii [AF355396]
OTU 812751
OTU 161302
Phormidium murrayii [AY493598]
DeNovo OTU51928
OTU 818024
Microcoleus steenstrupii [AF355379]
Microcoleus steenstrupii [AF355385]
Microcoleus steenstrupii [AF355381]
Microcoleus steenstrupii [AF355384]
OTU 277671
Microcoleus sociatus [AF284808]
Coleofasciculus chthonoplastes [KC463190]
OTU 278544
Microcoleus sociatus [AF284809]
Microcoleus steenstrupii [AF355380]
Microcoleus steenstrupii [AF355394]
OTU 278296
Microcoleus steenstrupii [AF355382]
OTU 649198
DeNovo OTU34989
Microcoleus steenstrupii [AJ871979]
OTU 551186
Microcoleus steenstrupii [AF355383]
DeNovo OTU44682
OTU 278136
Microcoleus paludosus [EF654090]
Microcoleus steenstrupii [AF355389]
Microcoleus steenstrupii [AF355386]
Microcoleus steenstrupii [AF355387]
Microcoleus steenstrupii [AF355388]
Microcoleus steenstrupii [AF355390]
Microcoleus steenstrupii [AF355391]
Microcoleus steenstrupii [AF355393]
DeNovo OTU761
Microcoleus steenstrupii [AF355392]
Microcoleus steenstrupii clade
0.07

Supplementary Figure 3: Phylogenetic assignment of the most abundant OTUs of *Microcoleus vaginatus* and *Microcoleus steenstrupii*

SSU rRNA gene maximun likelihood phylogenetic tree of the 13 most abundant Operational Taxonomic Units (OTUs) assigned to *Microcoleus steenstrupii* (comprising 97% to 100% of the sequences assigned to this taxon in each sample) and the 3 most abundant OTUs of *Microcoleus vaginatus* (comprising 91% to 100% of the sequences assigned to this species in each sample). Numbers at nodes indicate bootstrap values and the scale bar indicates the number of substitutions per site for a unit branch length. The out-group, not shown, is comprised of Gram + bacteria.

Supplementary Table 1

Summary of SSU rRNA gene sequences analyzed from M1-M5 BSC sample set and associated α-diversity indices.

Sample ID	Replicate	Total number of sequences	Total Number of OTU *	Number of singletons	Good's coverage (%)	Dataset rarefied at 13000 sequences per sample		
						Chao1	observed species	Phylogenetic distance (whole tree)
M1	B0	38426	1352	285	99.3	1297	986	89
M1	C0	13131	1074	316	97.6	1319	1067	95
M1	A1	28072	1199	404	98.6	1346	902	83
M1	B1	18836	1230	362	98.1	1421	1083	92
M1	C1	15981	1099	379	97.6	1444	1006	88
M1	A2	27982	1376	387	98.6	1475	1042	92
M1	B2	14892	1150	332	97.8	1406	1114	97
M1	C2	40279	1532	338	99.2	1438	1079	92
M2	A0	33435	1325	358	98.9	1343	986	91
M2	B0	79610	1856	294	99.6	1569	1166	102
M2	C0	67484	1761	321	99.5	1687	1128	100
M2	A1	37228	1319	345	99.1	1478	967	89
M2	B1	72781	1615	361	99.5	1429	956	90
M2	C1	63862	1541	384	99.4	1366	940	86
M2	A2	23910	1246	358	98.5	1365	1042	93
M2	B2	51408	1663	338	99.3	1621	1115	99
M2	C2	42113	1515	416	99.0	1645	1001	91
M3	A0	34834	1372	396	98.9	1368	975	92
M3	B0	43020	1493	393	99.1	1552	1037	96
M3	C0	43153	1341	343	99.2	1224	917	89
M3	A1	55582	1572	361	99.4	1374	1000	92
M3	B1	61600	1507	440	99.3	1326	865	84
M3	C1	54863	1592	390	99.3	1432	982	95
M3	A2	33844	1300	344	99.0	1324	960	91
M3	B2	29563	1334	384	98.7	1402	1015	94
M3	C2	48727	1467	353	99.3	1459	971	92
M4	A0	35043	1203	324	99.1	1362	869	85
M4	B0	37398	1456	405	98.9	1446	1019	97
M4	C0	86156	1698	326	99.6	1376	995	97
M4	A1	61779	1811	368	99.4	1732	1150	103
M4	B1	88569	1667	321	99.6	1372	950	94
M4	C1	52352	1519	430	99.2	1401	939	91
M4	A2	85716	1732	377	99.6	1425	961	91
M4	B2	28145	1310	371	98.7	1489	1039	96
M4	C2	42542	1477	376	99.1	1424	1012	97

M5	A0	63259	1625	297	99.5	1413	1021	89
M5	B0	66118	1711	326	99.5	1552	1043	95
M5	C0	51325	1430	360	99.3	1330	896	89
M5	A1	35755	1358	407	98.9	1347	939	85
M5	B1	106174	1871	375	99.6	1474	951	92
M5	C1	41717	1467	362	99.1	1491	1022	93
M5	B2	27333	1263	400	98.5	1425	974	90
M5	C2	46962	1471	383	99.2	1447	964	91

*OTU: group of sequences sharing >97% identity

Supplementary Table 2.

Repeated measures ANOVA on cyanobacterial proportion showing no effect of sampling time within sample

Sample ID	Scytonemin bearing Cyanobacteria (1)	*Microcoleus steenstrupii*	*Microcoleus vaginatus*
M1	0.136	0.377	0.217
M2	0.626	0.053*	0.092
M3	0.523	0.496	0.091
M4	0.774	0.911	0.414
M5	0.056*	0.768	0.38

(1) Including Nostoc, Scytonema and Tolypothrix. *In these cases post-hoc analyses were conducted, they were all >0.1 showing no interaction between time and cyanobacterial proportion. Overall there is no statistically supported effect of the sampling time on cyanobacterial proportion within this subset of samples.
