# Supplementary material for: Bacteria increase arid-land soil surface temperature through the production of sunscreens
Source: Nat Commun. 2016 Jan 20;7:10373. doi: 10.1038/ncomms10373 (PMC4735820; doi:10.1038/ncomms10373)
Supplement: Supplementary Software — Parameter file used for the treatment of Illumina 16S rDNA libraries data in the qiime pipeline. [file ncomms10373-s2.docx]

**Parameter file used for the treatment of Illumina 16S rDNA libraries data in the qiime pipeline**

# qiime_parameters.txt

# OTU picker parameters

pick_otus:otu_picking_method uclust_ref

pick_otus:similarity 0.97

pick_otus:refseqs_fp /home/ubuntu/gg_12_10/gg_97_12_10_V4.fasta

pick_otus:enable_rev_strand_match True

# Parallel options

parallel:jobs_to_start 1

parallel:retain_temp_files False

parallel:seconds_to_sleep 60

# Representative set picker parameters

pick_rep_set:rep_set_picking_method most_abundant

pick_rep_set:sort_by otu

# Multiple sequence alignment parameters

align_seqs:template_fp /home/ubuntu/gg_12_10/gg_97_12_10_aligned_V4.fasta

align_seqs:alignment_method pynast

align_seqs:pairwise_alignment_method uclust

align_seqs:min_length 75

align_seqs:min_percent_id 75.0

# Alignment filtering (prior to tree-building) parameters

filter_alignment:lane_mask_fp /home/ubuntu/gg_12_10/lanemask_in_1s_and_0s_V4.fasta

filter_alignment:allowed_gap_frac 0.999999

filter_alignment:remove_outliers False

filter_alignment:threshold 3.0

# Taxonomy assignment parameters

assign_taxonomy:id_to_taxonomy_fp /home/ubuntu/gg_12_10/97_otu_taxonomy.txt

assign_taxonomy:reference_seqs_fp /home/ubuntu/gg_12_10/gg_97_12_10_V4.fasta

assign_taxonomy:assignment_method rdp

assign_taxonomy:confidence 0.7

# Phylogenetic tree building parameters

make_phylogeny:tree_method fasttree

make_phylogeny:root_method tree_method_default

# Beta diversity parameters

beta_diversity:metrics weighted_unifrac,unweighted_unifrac

# Make 3D plot parameters

make_3d_plots:ellipsoid_smoothness 1

# Rarefaction parameters

multiple_rarefactions:num-reps 10

multiple_rarefactions:lineages_included False

# Even-depth rarefaction parameters

multiple_rarefactions_even_depth:num-reps 100

# Alpha diversity parameters

alpha_diversity:metrics PD_whole_tree,chao1,observed_species

# Make rarefaction plots parameters

make_rarefaction_plots:imagetype png

make_rarefaction_plots:resolution 75

make_rarefaction_plots:background_color white

make_rarefaction_plots:prefs_path
